# Bioinformatic analysis of allergens

**DOI:** 10.1186/2045-7022-1-S1-S22

**Published:** 2011-08-12

**Authors:** Steven Gendel

**Affiliations:** 1 Food and Drug Administration, Center for Food Safety and Applied Nutrition, Office of Food Additive Safety, Maryland, USA

## 

The number of resources containing data on the sequence, structure, and evolution of allergenic proteins has increased significantly in the last several years. At the same time, informatics resources related to the functioning of the immune system continue to mature rapidly. This proliferation creates a need for effective means to locate, extract, and integrate data from multiple sources to advance our understanding of allergens and allergy. Effective data integration rests on the development and application of both a system of data descriptors (metadata) and methods for data exchange between information repositories and analytic resources. A well designed metadata system for allergens should include descriptors for primary data, annotations, and data manipulation tools. An effective system would also allow integration with electronic clinical, nutrition, labeling, and health resources. The process of developing such a system for allergens creates an opportunity to identify and formalize important concepts that are widely used but often poorly defined. For example, formal terminology could be developed such that allergen databases could include descriptors for the evidence used to identify epitope sequences or cleavage sites. An XML schema called AllerML has been described that demonstrates the implementation of a terminology system for allergen data. One of the benefits inherent in using a system of shared data descriptors is that database developers can use it as the basis for implementing “application program interfaces” (APIs) that permit analysis software to directly access data within a database. The essential next steps in the creation of an integrated bioinformatics system for allergens based on currently existing opportunities and resources will be described.

